# Ultrasound Imaging and Guidance for Distal Peripheral Nerve Pathologies at the Wrist/Hand

**DOI:** 10.3390/diagnostics13111928

**Published:** 2023-05-31

**Authors:** Wei-Ting Wu, Ke-Vin Chang, Yu-Chun Hsu, Yuan-Yuan Tsai, Kamal Mezian, Vincenzo Ricci, Levent Özçakar

**Affiliations:** 1 Department of Physical Medicine and Rehabilitation, National Taiwan University Hospital, Bei-Hu Branch, Taipei 10845, Taiwan; wwtaustin@yahoo.com.tw (W.-T.W.); viph062@gmail.com (Y.-C.H.); pmrdrtsai@gmail.com (Y.-Y.T.); 2 Department of Physical Medicine and Rehabilitation, National Taiwan University College of Medicine, Taipei 10048, Taiwan; 3 Center for Regional Anesthesia and Pain Medicine, Wang-Fang Hospital, Taipei Medical University, Taipei 11600, Taiwan; 4 Department of Rehabilitation Medicine, First Faculty of Medicine and General University Hospital, Charles University, 12800 Prague, Czech Republic; kamal.mezian@gmail.com; 5 Physical and Rehabilitation Medicine Unit, Luigi Sacco University Hospital, ASST Fatebenefratelli-Sacco, 20157 Milan, Italy; vincenzo.ricci58@gmail.com; 6 Department of Physical and Rehabilitation Medicine, Hacettepe University Medical School, Ankara 20157, Turkey; lozcakar@yahoo.com

**Keywords:** ultrasonography, neuropathy, entrapment, diagnosis, finger

## Abstract

Ultrasound has emerged as a highly valuable tool in imaging peripheral nerve lesions in the wrist region, particularly for common pathologies such as carpal tunnel and Guyon’s canal syndromes. Extensive research has demonstrated nerve swelling proximal to the entrapment site, an unclear border, and flattening as features of nerve entrapments. However, there is a dearth of information regarding small or terminal nerves in the wrist and hand. This article aims to bridge this knowledge gap by providing a comprehensive overview concerning scanning techniques, pathology, and guided-injection methods for those nerve entrapments. The median nerve (main trunk, palmar cutaneous branch, and recurrent motor branch), ulnar nerve (main trunk, superficial branch, deep branch, palmar ulnar cutaneous branch, and dorsal ulnar cutaneous branch), superficial radial nerve, posterior interosseous nerve, palmar common/proper digital nerves, and dorsal common/proper digital nerves are elaborated in this review. A series of ultrasound images are used to illustrate these techniques in detail. Finally, sonographic findings complement electrodiagnostic studies, providing better insight into understanding the whole clinical scenario, while ultrasound-guided interventions are safe and effective for treating relevant nerve pathologies.

## 1. Introduction

**Figure 1:**
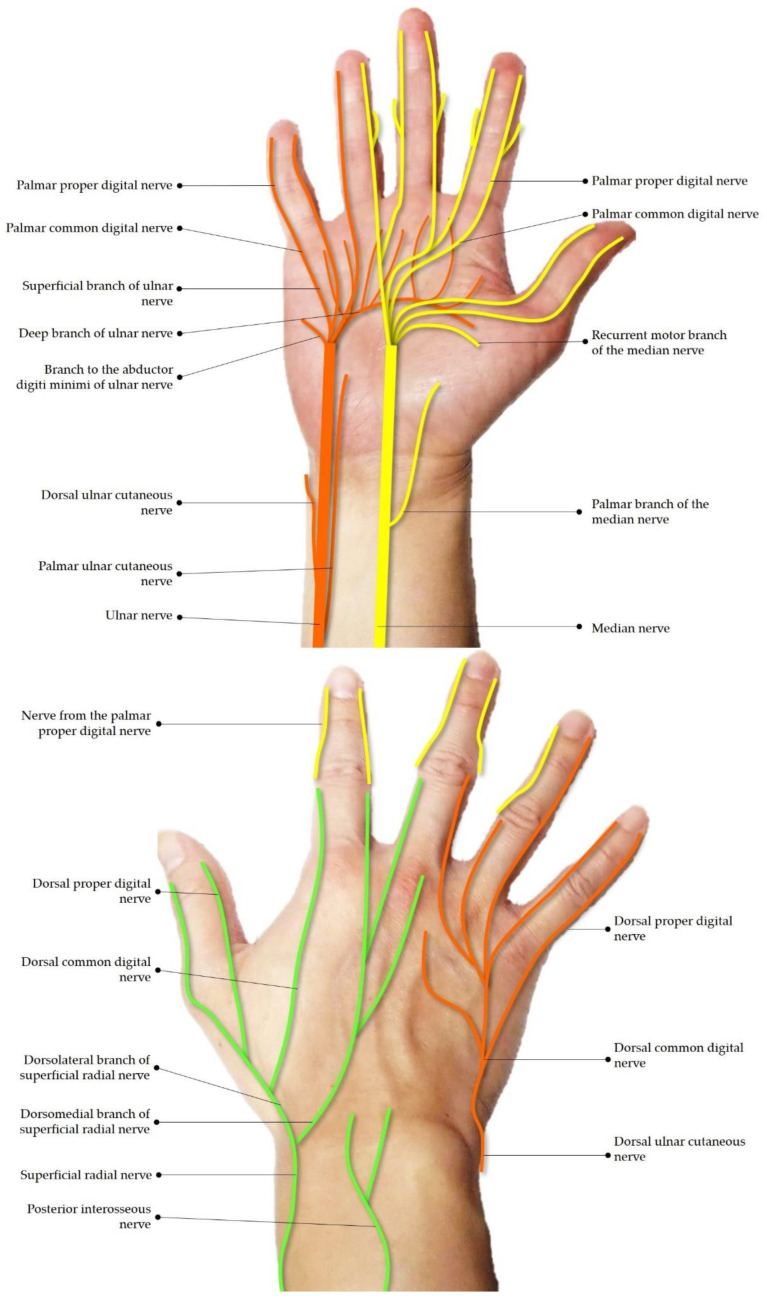
Schematic drawing of the innervation of the wrist and hand.

While magnetic resonance imaging (MRI) can be used to assess peripheral nerve pathologies, high cost and lack of portability limit its widespread use in clinical settings. Additionally, MRI cannot be used to guide injections for entrapped nerves. In recent years, high-resolution ultrasound (US) has increasingly been used to investigate peripheral nerve lesions, with its diagnostic performance comparable to MRI [1,2]. Although numerous clinical guidelines have addressed scanning techniques for peripheral nerves [1,3,4,5,6], they mainly focused on the proximal/main nerve trunks [2,7,8]. There are few pictorial reviews targeting terminal nerves in the wrist and hand. Furthermore, most existing literature emphasizes carpal tunnel [9] and Guyon’s canal syndromes [10], while other nerve entrapment syndromes, such as compression of the recurrent motor branch of the median nerve, are rarely mentioned. Herein, this pictorial essay aims to provide a detailed overview of regional neural anatomy (Figure 1), scanning skills, and sonographic pathologies for the main nerves and their distal branches at the wrist and hand. Of note, several US images and videos are illustrated for prompt diagnosis and guided injections of pertinent nerve entrapment syndromes [11,12].

## 2. General Considerations

### 2.1. Ultrasound Setting and Scanning Plane

In the present pictorial review, high-frequency linear transducers (5–18 MHz; HI VISION, Ascendus, Hitachi, Japan, and Aplio 500, Canon, Tokyo, Japan) were used for scanning the nerves. The transducer was placed in the nerve’s short axis to delineate its epineurium during quantification of its cross-sectional area (CSA) [13,14,15]. Herewith, the transducer was positioned along the nerve’s long axis for prompt identification of the entrapped segment. Additionally, Doppler imaging was used to detect accompanying vessels or peri/intraneural hypervascularity due to inflammatory pathologies.

### 2.2. Common Ultrasonographic Features of Peripheral Nerve Pathologies

Common causes of peripheral nerve lesions are entrapment [11,12], tumors, and trauma. Nerve compression can obstruct microvasculature, leading to Schwann cell necrosis and subsequent demyelination. Severe cases can result in arterial flow obstruction, axonal injury, and degeneration, which can be detected in electrophysiological examinations as well. Duration and magnitude of nerve compression are directly related to the severity of symptoms. US can identify swollen nerve fascicles proximal and distal to the site of compression [16]. It is also useful in identifying the underlying cause of entrapment, e.g., hypertrophic scar.

Primary peripheral nerve tumors are mostly benign, such as schwannoma and neurofibroma. The former originates from Schwann cells within the neural sheath and appears as an encapsulated hypoechoic mass (with a round, fusiform, or oval shape), which can cause compression and lateral displacement of the nerve trunk [17,18]. On the other hand, neurofibromas are commonly associated with neurofibromatosis type 1 and display a mixed origin, including Schwann cells, fibroblasts, mast cells, and axons [19,20]. Neurofibromas usually have a hypoechoic, fusiform, and lobulated shape on US, with significantly larger diameters than that of schwannomas [21]. In contrast, a malignant peripheral nerve tumor tends to be larger in size, with ill-defined margins, central necrosis, and infiltration of the surrounding tissues [22].

The mechanisms of traumatic nerve injuries include compression, traction, crush, and transection. Traction and compression injuries are usually associated with neurapraxia, presenting as focal demyelination without damage to the axons or connective tissues. A crush injury often results from contusion by a blunt object (e.g., bat or surgical clamp) and can cause varying degrees of neural damage [23]. On the other hand, a transection injury caused by a glass shard, knife, or gunshot typically leads to neurotmesis, which involves complete discontinuation of the nerve [23]. US features of traumatic nerve injuries may include nerve swelling (i.e., enlarged CSA), loss of normal architecture, disruption of the fascicular pattern, and loss of nerve continuity [24].

To establish a definitive diagnosis of a peripheral nerve pathology, it is crucial to conduct a comparative analysis of US findings in the affected vs. contralateral side. It is noteworthy that sono-Tinel sign, which involves using the transducer to compress the target nerve and elicit symptoms, can aid in precisely locating the lesion. Moreover, the related muscles become atrophied/hyperechoic as a result of denervation [25].

### 2.3. Ultrasound-Guided Hydrodissection

A diagnostic US-guided nerve block with local anesthetics can be performed to confirm the clinical scenario. Hydrodissection (using regimens such as 5% dextrose or corticosteroid) is suggested to first target the nerve’s short axis for better delineating the epineurium [9,26,27]. The needle tip can be placed with its bevel up for the upper half of the epineurium and bevel down for the lower half of the epineurium, to fully separate the epineurium from the surrounding connective tissues. In the case of a long-segment entrapment, an alternative approach would be to align the transducer with the target nerve’s long axis for hydrodissecting the upper half of the epineurium.

## 3. Sonoanatomy and Pathology

### 3.1. Median Nerve

#### 3.1.1. Main Trunk

##### Anatomy

The median nerve originates from the lateral and medial cords of the brachial plexus, which are formed by the ventral roots from C5 to T1. Apart from the flexor carpi ulnaris and the ulnar half of the flexor digitorum profundus, the median nerve provides innervation to the anterior compartment of the forearm muscles. At the palmar wrist, the nerve innervates the first and second lumbrical muscles and thenar muscles. In terms of sensory innervation, it supplies the palmar surface of the thumb and the second, third, and radial half of the fourth fingers.

##### Scanning Technique

**Figure 2:**
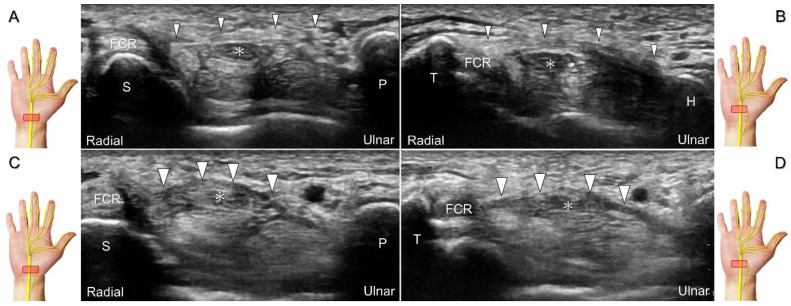
Sonographic/normal imaging of the median nerve from the inlet (**A**) to the outlet (**B**) of the carpal tunnel. Hypertrophy of the flexor retinaculum at both the inlet (**C**) and outlet (**D**) of the carpal tunnel. Asterisk: median nerve; small white arrowheads: normal flexor retinaculum; large arrowheads: hypertrophy of the flexor retinaculum. FCR: flexor carpi radialis; S: scaphoid; P: pisiform; T: trapezium; H: hook of the hamate.

The transducer can be placed in the axial plane on the distal one-third of the forearm with the forearm supinated. The median nerve travels between the flexor digitorum superficialis and flexor digitorum profundus muscles. It gradually runs superficially to enter the carpal tunnel. The carpal tunnel inlet is defined as the plane crossing the scaphoid and pisiform (Figure 2A), whereas the plane linking the trapezium and hook of the hamate serves as the carpal tunnel outlet (Figure 2B).

##### Clinical Implication

**Figure 3:**
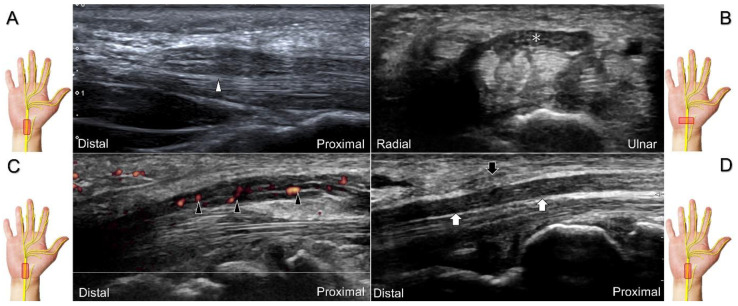
Sonographic images of patients with carpal tunnel syndrome, showing focal swelling proximal to the compression site (**A**), flattening at the compression site (**B**), intraneural hypervascularity (**C**), and loss of the trimline pattern (**D**). White arrowhead: focal swelling of the median nerve; asterisk: flattening of the median nerve; black arrowheads: intraneural hypervascularity of the median nerve; white arrows: loss of the trimline pattern of the median nerve; black arrow: thickened flexor retinaculum.

Carpal tunnel syndrome is the most common entrapment neuropathy whereby the median nerve is entrapped by various causes, like hypertrophy of the flexor retinaculum (Figure 2C,D) and compression from the accessory muscles, swollen tendons, ganglions, and bony fractures within the tunnel. Ultrasonographic changes encompass swelling proximal to the entrapment site (Figure 3A), flattening over the entrapment site (Figure 3B), intraneural hypervascularity (Figure 3C), and focal loss of the trimline pattern (Figure 3D).

**Figure 4:**
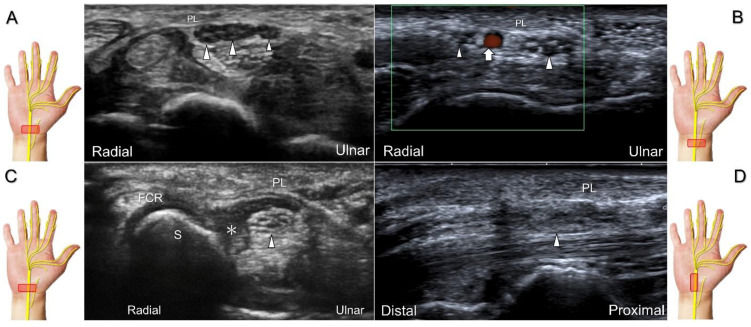
Sonographic images show a bifid median nerve (**A**), a persistent median artery with a bifid median nerve (**B**), accessory flexor digitorum superficialis muscle (**C**), and laceration of the palmaris longus (PL) tendon with the compression of the median nerve (**D**). White arrowheads: median nerve; white arrow: persistent median artery; asterisk: accessory flexor digitorum superficialis muscle. FCR: flexor carpi radialis tendon; S: scaphoid.

**Figure 5:**
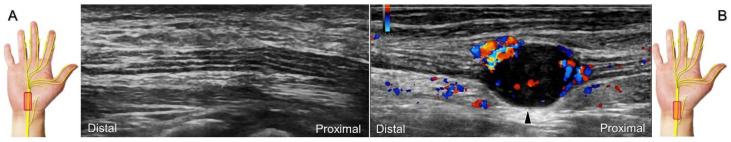
Comparative ultrasonography (long-axis view) between healthy (**A**) vs. affected (**B**) sides shows a schwannoma (black arrowhead) with increased intraneural vascularity.

The nerve’s CSA (a cutoff value of 9–10.5 mm^2^) arises as the most useful parameter for the diagnosis [28], whereas its diameter, gliding resistance [29], stiffness (evaluated by sonoelastography), and intraneural vascularity (assessed by power Doppler imaging) may serve as adjuvant indicators. A bifid median nerve (Figure 4A), the presence of a persistent median artery (Figure 4B) with or without thrombosis, accessory flexor digitorum superficialis muscle (Figure 4C), laceration of the palmaris longus tendon (Figure 4D), and schwannoma (Figure 5) can be associated findings for carpal tunnel syndrome [9].

**Figure 6:**
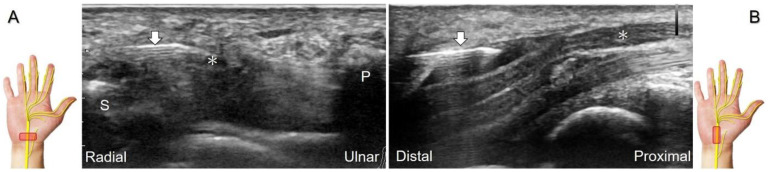
Ultrasound-guided hydrodissection of the median nerve under short-axis (**A**) or long-axis (**B**) imaging. Asterisk: median nerve; arrows: needle. S: scaphoid; P: pisiform.

Regarding US-guided injections for carpal tunnel syndrome, a network meta-analysis, including ten studies with 497 patients, reported that 5% dextrose (D5W) injection was likely to be the best treatment for symptom relief, followed by platelet-rich plasma injection [30]. During hydrodissection of the median nerve, the needle can be introduced using either the in-plane approach targeting the short-axis (Figure 6A, Video S1) or long-axis (Figure 6B, Video S2) views of the nerve. If symptoms persist despite non-operative management, consideration should be given to minimal invasive carpal tunnel release, with confirmation of complete release using dynamic US [31].

#### 3.1.2. Palmar Cutaneous Branch of the Median Nerve

##### Anatomy

The palmar cutaneous branch of the median nerve is responsible for sensory innervation to the skin of the thenar and proximal palmar regions. It originates from the radial side of the median nerve in the distal forearm, perforates the antebrachial fascia between the flexor carpi radialis and palmaris longus tendons, and does not enter the carpal tunnel. The nerve then divides into the medial and lateral branches distal to the flexor retinaculum, providing cutaneous sensation to the palm and the ball of the thumb.

##### Scanning Technique

**Figure 7:**
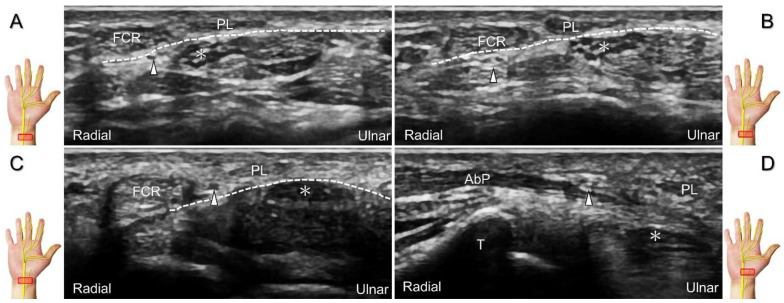
Sonographic imaging of the palmar cutaneous branch of the median nerve (**A**) shows its emerging from the radial aspect of the median nerve (**B**), penetrating the antebrachial fascia (**C**), and arriving at the superficial site of the abductor pollicis brevis muscle (**D**). Asterisk: median nerve; arrowhead: palmar cutaneous branch of the median nerve; dashed line, antebrachial fascia; FCR: flexor carpi radialis tendon; T: trapezium; PL: palmaris longus tendon; AbP: abductor pollicis brevis muscle.

The transducer is first placed in the axial plane over the distal forearm with the forearm supinated to locate the median nerve between the flexor digitorum superficialis and profundus muscles (Figure 7A). Moving the transducer distally, the palmar cutaneous branch of the median nerve, shown as a single hypoechoic fascicle, emerges from the radial aspect of the median nerve (Figure 7B) [32]. The nerve then penetrates the antebrachial fascia and runs on the ulnar aspect of the flexor carpi radialis tendon (Figure 7C). Eventually, the nerve can be identified superficial to the abductor pollicis brevis muscle (Figure 7D).

##### Clinical Implication

**Figure 8:**
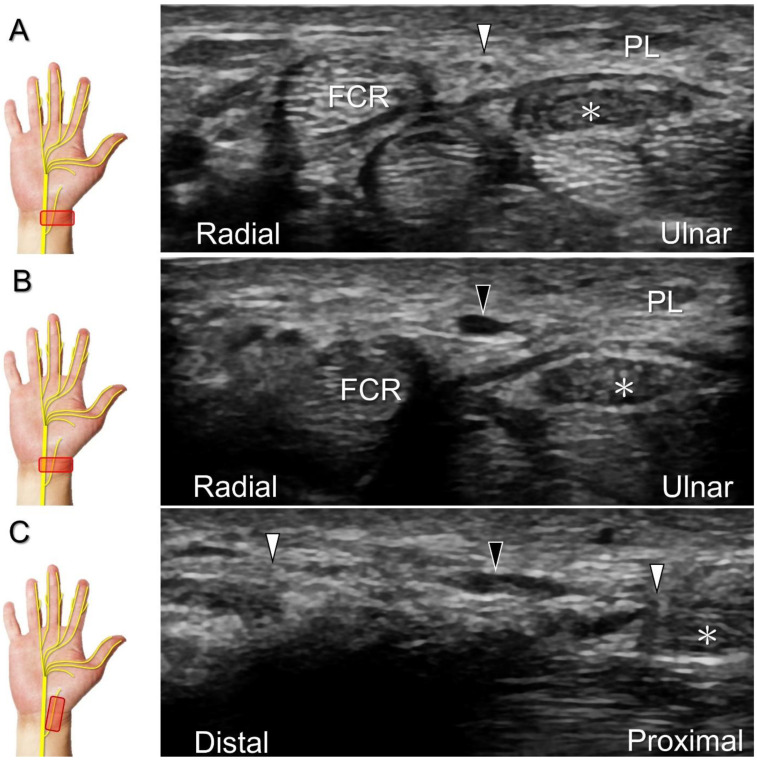
Compared with the normal proximal segment (**A**), a neuroma originating from the palmar cutaneous branch of the median nerve is seen in short-axis (**B**) and long-axis (**C**) imaging. Asterisk: median nerve; white arrowheads: palmar cutaneous branch of the median nerve; black arrowhead: neuroma. FCR: flexor carpi radialis tendon; PL: palmaris longus tendon.

**Figure 9:**
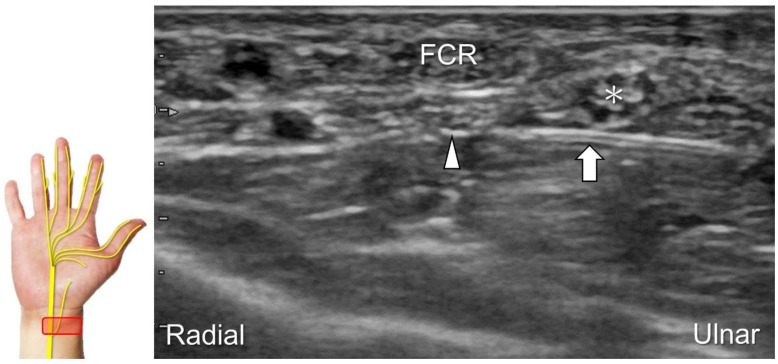
Ultrasound-guided hydrodissection (short-axis view) for the palmar cutaneous branch of the median nerve. Asterisk: median nerve; arrowhead: palmar cutaneous branch of the median nerve; arrow: needle. FCR: flexor carpi radialis tendon.

The palmar cutaneous branch of the median nerve can be injured due to cuts, resulting in neuroma formation (Figure 8A–C) [9]. During an intervention for carpal tunnel syndrome, it is essential to identify the palmar cutaneous branch of the median nerve to prevent accidental injury. Recurrent pain and paresthesia over the palmar region following carpal tunnel release can also be subsequent to entrapment by post-operative scar tissues. For hydrodissection of this nerve, the preferred approach is an in-plane approach targeting its short axis from the ulnar aspect (Figure 9).

#### 3.1.3. Recurrent Motor Branch of the Median Nerve

##### Anatomy

The recurrent motor branch of the median nerve innervates the thenar muscles, i.e., abductor pollicis brevis, opponens pollicis, and the superficial head of the flexor pollicis brevis. There are four main types of reciprocal anatomy between this branch and the transverse carpal ligament. The mean diameter of the recurrent motor branch has been reported as 0.7 mm—with a standard deviation of 0.1 mm in healthy volunteers [33]. The extraligamentous type is the most common [34,35], where the nerve emerges from the main trunk distal to the carpal tunnel outlet, ascends vertically to the palmar surface through the distal edge of the transverse carpal ligament [36], and then curves back to innervate the thenar muscles. However, if the recurrent motor branch exits the main trunk in other locations, the nerve is classified as preligamentous, subligamentous, or transligamentous, depending on whether it exits proximal to the flexor retinaculum, within the carpal tunnel, or by piercing the transverse carpal ligament, respectively [35].

##### Scanning Technique

**Figure 10:**
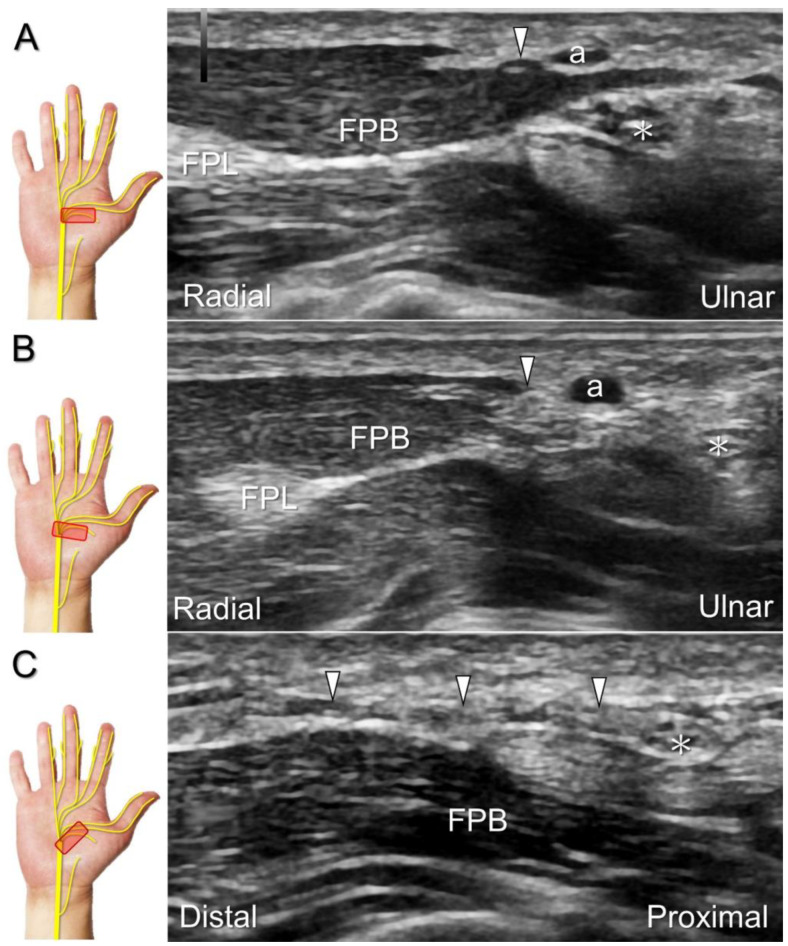
Sonographic imaging for the recurrent motor branch of the median nerve. Tracking back from the surface of the thenar muscle (**A**) toward the division site from the main trunk in short-axis (**B**) and long-axis (**C**) imaging. Asterisk: median nerve; white arrowheads: recurrent motor branch of the median nerve; a: artery; FPL: flexor pollicis longus tendon; FPB: flexor pollicis brevis muscle.

**Figure 11:**
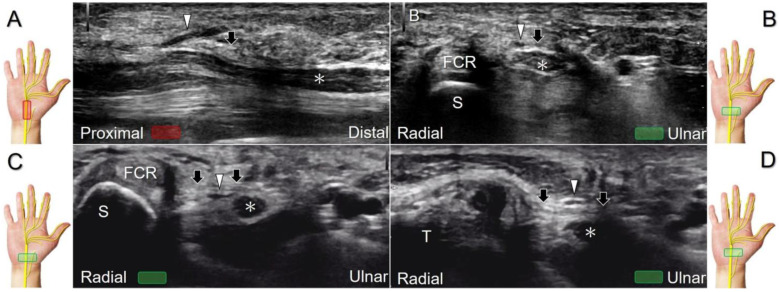
Sonographic imaging of the preligamentous type of recurrent motor branch of the median nerve. It divides from the median nerve proximal to the carpal tunnel as seen in the long-axis view (**A**), and resides superficial to the flexor retinaculum, as seen in the short-axis view (**B**), whereas the transligamentous type of recurrent motor branch accompanies the median nerve into the carpal tunnel (**C**), penetrating the retinaculum at the outlet of the carpal tunnel (**D**). Asterisk: median nerve; arrowheads: recurrent motor branch of the median nerve; arrows: flexor retinaculum. FCR: flexor carpi radialis tendon.

To assess the median nerve and its branches, the transducer is initially placed over the carpal tunnel inlet and then moved distally/radially. In the extraligamentous type, the recurrent motor branch emerges from the radial aspect of the median nerve as it travels distally to the carpal tunnel outlet. However, at this point, identifying the precise site of branching may be challenging due to anisotropy and the nerve’s reversed course. To aid visualization, the physician can pivot the radial end of the transducer back and forth to identify the recurrent motor branch. It appears as a hypoechoic monofascicle that pierces the palmar aponeurosis, reaching the surface of the thenar muscle (Figure 10). Occasionally, other anatomic variants can also be visualized (Figure 11) [37].

##### Clinical Implication

**Figure 12:**
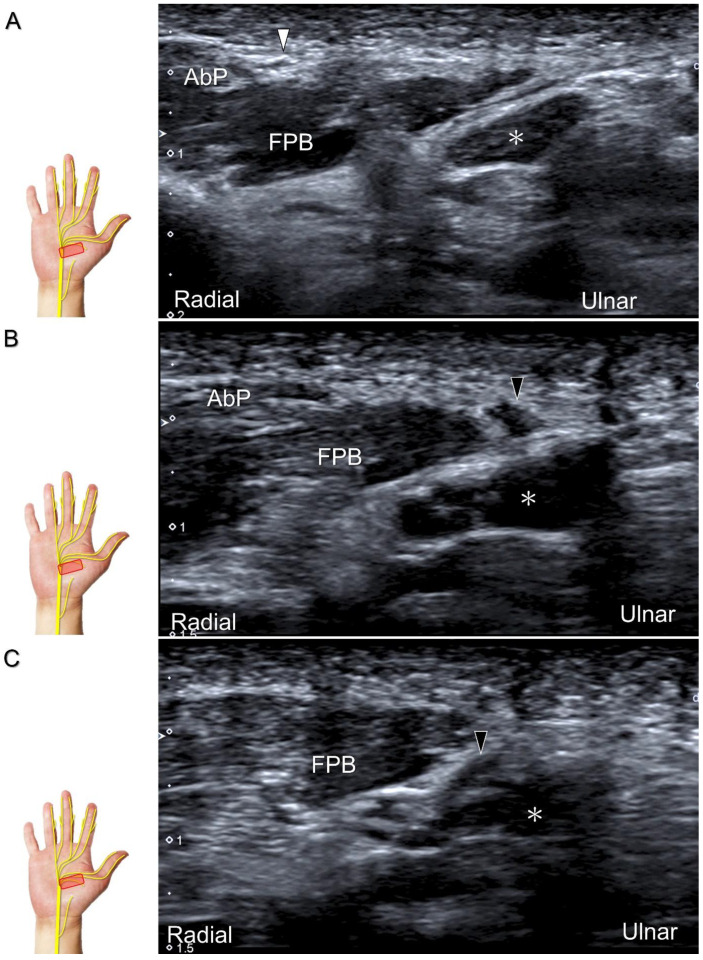
Sonographic imaging (short-axis view) shows the segment (**A**) distal to the neuroma of the recurrent motor branch of the median nerve (**B**) and the proximal segment (**C**). Asterisk: median nerve; white arrowhead: recurrent motor branch of the median nerve; black arrowheads: neuroma. FPB: flexor pollicis brevis muscle; AbP: abductor pollicis brevis muscle.

The recurrent motor branch may be injured during carpal tunnel release, trigger point injection of the thenar muscle, and repetitive impact of the thenar eminence [34]. In the case of nerve injury, the thenar muscle is likely to undergo atrophy, leading to a subsequent reduction in pinch and grasp forces. US reveals a swollen fascicle and an indistinct border of the injured nerve, with poor visualization of the epineurium (Figure 12; Video S3). US-guided hydrodissection in short-axis view has been reported helpful for reducing thenar pain in a case with recurrent motor branch entrapment [36].

### 3.2. Ulnar Nerve

#### 3.2.1. Main Trunk, Superficial, and Deep Branches

##### Anatomy

The ulnar nerve originates from the medial cord of the brachial plexus, specifically from the C8 and T1 nerve roots. As it approaches the wrist region, it courses below the flexor carpi ulnaris (FCU) tendon alongside the ulnar artery. It then enters the Guyon’s canal, bordered by the palmar carpal ligament, transverse carpal ligament, and pisiform. Beyond the canal, a concave fibrous arch connects the two attachments of the flexor digiti minimi brevis muscle on the flexor retinaculum and the hamate hook [38]. The fibrous arch constitutes the roof of the narrow pisohamate hiatus, whereby its floor is formed by the pisohamate ligament [39].

The ulnar nerve gives rise to two terminal (i.e., superficial and deep) branches, which are divided from the main trunk prior to the hook of hamate. The superficial branch provides sensory innervation to the volar and dorsal regions of the little finger and the ulnar half of the ring finger, and motor innervation to the palmaris brevis muscle. The deep branch, accompanied by an artery, passes through the pisohamate hiatus and courses between the flexor digiti minimi brevis and opponens digiti minimi muscles. It subsequently enters the deep palm alongside the deep palmar branch of the ulnar artery, supplying the abductor digiti minimi, flexor digiti minimi brevis, opponens digiti minimi, deep head of flexor pollicis brevis, adductor pollicis, and the two lumbrical muscles located on the ulnar aspect and the palmar/dorsal interossei muscles.

##### Scanning Technique

**Figure 13:**
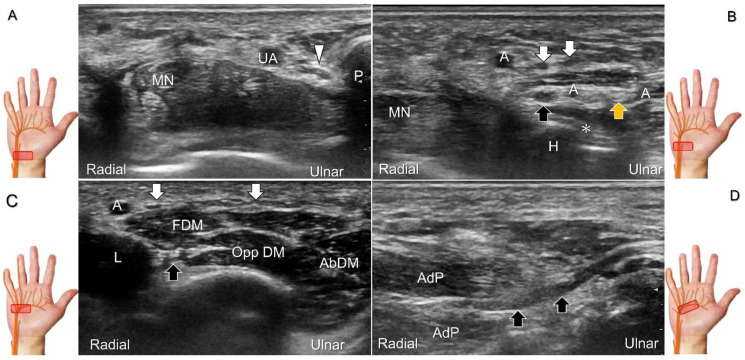
Sonographic imaging (short-axis view) shows the ulnar nerve within the Guyon’s canal (**A**), separation of branches beside the pisohamate hiatus (**B**), deep branch of the ulnar nerve located between the hypothenar muscles distal to the hook of the hamate (**C**). Long-axis view (through pivoting the transducer) shows the segment of the deep branch within the adductor pollicis muscle (**D**). Arrowhead: ulnar nerve; white arrows: superficial branch of the ulnar nerve; black arrows: deep branch of the ulnar nerve; orange arrow: branch of the ulnar nerve to the abductor digiti minimi; *: pisohamate ligment MN: median nerve; UA: ulnar artery; P: pisiform; H: hook of hamate; A: artery; L: lumbrical muscle; FDM: flexor digiti minimi brevis muscle; Opp DM: opponens digiti minimi muscle; AbDM: abductor digiti minimi muscle; AdP: adductor pollicis muscle.

To locate the Guyon’s canal, the transducer is placed along the axial plane on the volar wrist with the forearm supinated. This will reveal the ulnar nerve and artery within the canal (Figure 13A). To investigate the nerve’s long axis, the transducer is rotated 90 degrees. By moving the transducer toward the finger, the superficial and deep branches can be seen. Between the pisiform and hamate, the superficial and deep branches (along with their accompanying vessels) can be visualized as separated by the fibrous arch of hypothenar muscles, respectively (Figure 13B) [39].

The superficial branch divides into two palmar digital nerves. They run superficially along the little finger and half of the ring finger. The deep branch, on the other hand, can be observed between the flexor digiti minimi brevis and opponens digiti minimi muscles before it penetrates the deep aspect of the palm (Figure 13C). By pivoting the transducer, the physician can display the long axis of the deep branch, extending all the way to the segment within the adductor pollicis muscle (Figure 13D).

##### Clinical Implication

Injuries to the ulnar nerve beyond the elbow can cause claw hand, which manifests as hyperextension at the metacarpophalangeal joints and flexion at the proximal and distal interphalangeal joints of the fourth and fifth fingers—due to the unopposed action of the ulnar side of the flexor digitorum profundus muscle against the paralyzed fourth and fifth lumbrical muscles. If the nerve injury is distal to the wrist crest, it can lead to Guyon’s canal syndrome or ulnar tunnel syndrome. To investigate such cases, the mean pooled cross-sectional area (CSA) of the ulnar nerve at the Guyon’s canal in healthy volunteers (4.1 mm^2^ with a 95% CI between 3.6 and 4.6 mm^2^) can be used as a reference [40].

**Figure 14:**
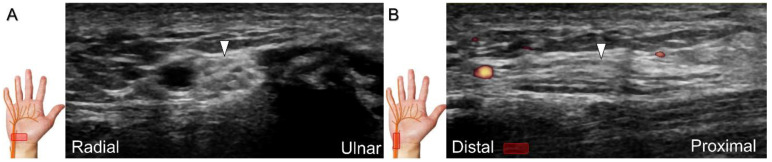
Sonographic images depict a fibrolipomatous hamartoma (arrowheads) in the short- (**A**) and long-axis (**B**) views.

**Figure 15:**
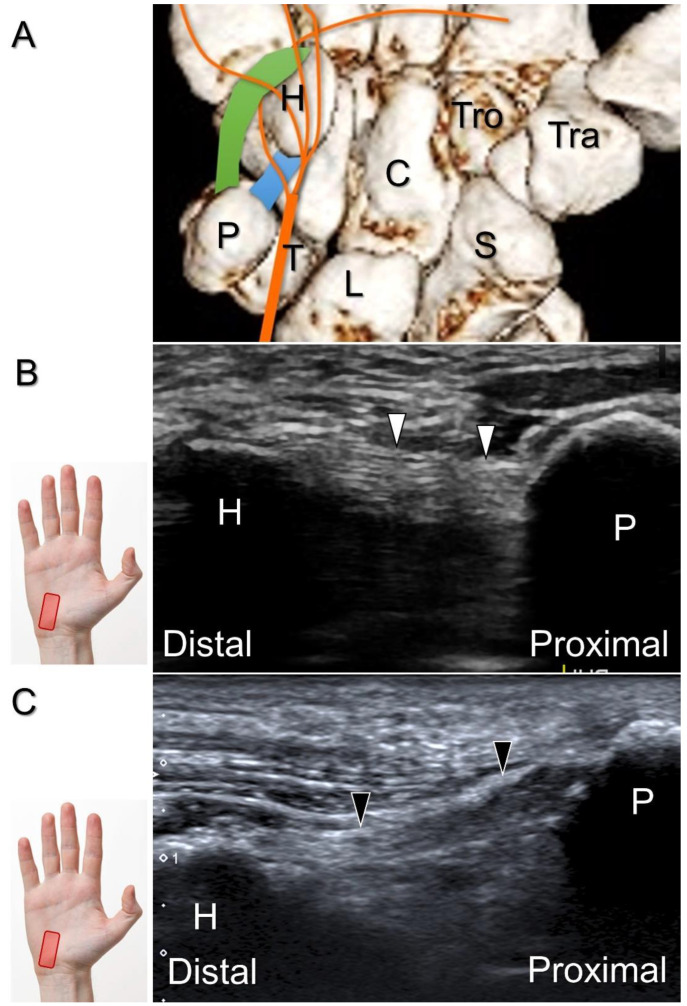
Illustration of the pisohamate hiatus formed by the arch of hypothenar muscles and the pisohamate ligament (**A**). Compared with the normal ligament (**B**), long-axis imaging displays sprain and swelling of the pisohamate ligament (**C**). Blue area: pisohamate ligament; green area: fibrous arch of the flexor digiti minimi brevis muscle; white arrows: normal pisohamate ligament; black arrows: swollen pisohamate ligament; H: hamate; P: pisiform; T: triquetrum; L: lunate; C: capitate; Tro: trapezoid; Tra: trapezium; S: scaphoid.

Repeat contusions to the hypothenar region and space-occupying lesions such as ganglion cysts, fracture segments, spurs of the pisiform, schwannoma, ulnar artery thrombosis, or aneurysms and fibrolipomatus hamatoma (Figure 14) can cause Guyon’s canal syndrome. Compression at the inlet of the Guyon’s canal, which is located proximal to the bifurcation of the ulnar nerve into the superficial and deep branches, can result in both sensory and motor deficits. However, if the injury is located more distally (Figure 15A), e.g., due to a fracture of the hamate or pisohamate ligament sprain (Figure 15B,C), only motor deficits may be observed [38].

**Figure 16:**
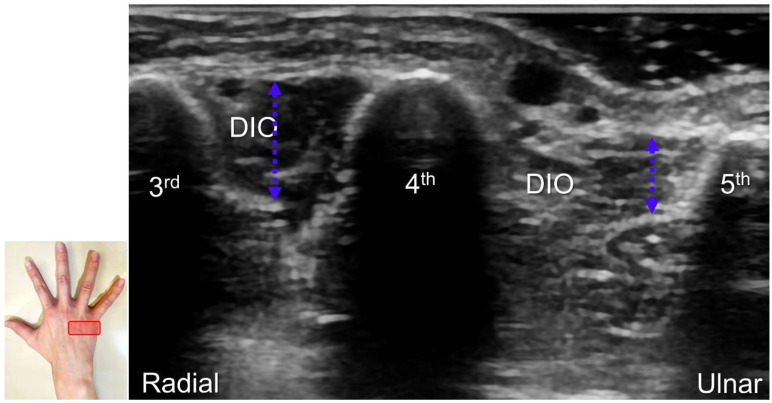
As opposed to the normal third dorsal interossei (DIO) muscle, atrophy in the fourth DIO muscle indicates injury to the terminal branch of the deep branch of the ulnar nerve. Blue double dashed arrows: muscle thickness.

**Figure 17:**
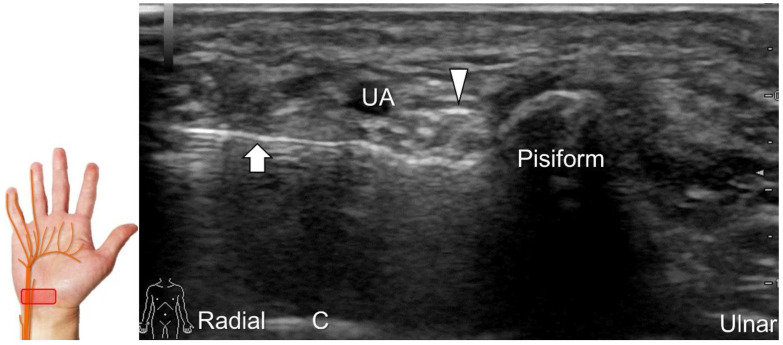
Ultrasound-guided injection of the ulnar nerve in short-axis view. Arrowhead: ulnar nerve; arrow: needle. UA: ulnar artery; C: capitate.

Handlebar neuropathy is a similar scenario that occurs due to continuous compression of the ulnar nerve at the ulnar wrist in cyclists. Focal swelling of the deep branch can be seen at the hamate level. An associated finding would be atrophy/weakness of the dorsal interossei muscles (Figure 16). For treatment, injection over the short axis of the ulnar nerve can be performed using the in-plane approach from the radial aspect in the Guyon’s canal (Figure 17). For certainty, identifying the ulnar artery is essential to prevent iatrogenic injury.

#### 3.2.2. Palmar Ulnar Cutaneous Nerve

##### Anatomy

The palmar ulnar cutaneous nerve is a distal branch of the ulnar nerve and it originates from the main trunk approximately 5 to 10 cm proximal to the wrist crease. A cadaveric study revealed that the nerve has a mean diameter of 0.8 mm [41]. Together with the ulnar nerve and vessels, the nerve courses below the flexor carpi ulnaris (FCU) tendon. It enters the Guyon’s canal and perforates the palmar carpal ligament. It then courses between the palmaris longus and FCU tendons, and in some cases, communicates with the superficial and deep branches of the ulnar nerve, and even with the palmar cutaneous branch of the median nerve [41]. The nerve is responsible for supplying sensation to the hypothenar eminence and carries a vascular branch to the ulnar artery.

##### Scanning Technique

**Figure 18:**
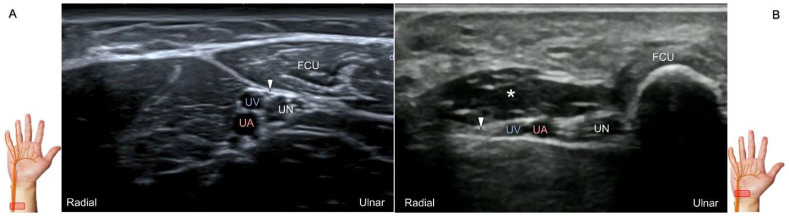
Sonographic imaging (short-axis view) of the palmar ulnar cutaneous nerve (**A**) and its entrapment in the presence of an accessory abductor digiti minimi muscle (**B**). Arrowhead: palmar ulnar cutaneous nerve; asterisk: accessory abductor digiti minimi muscle. UN: ulnar nerve; UA: ulnar artery; UV: ulnar vein; FCU: flexor carpi ulnaris tendon.

During scanning, the forearm is supinated with the transducer placed along the axial plane of the distal forearm. Moving the transducer more distally, the palmar ulnar cutaneous nerve can be seen as a single hypoechoic fascicle departing from the radial side of the main trunk near the bifurcating point for the dorsal ulnar cutaneous nerve (Figure 18A). It is crucial to apply light touch without compressing the adjacent vein, which can be used to highlight the nerve’s border.

##### Clinical Implication

The palmar ulnar cutaneous nerve has been associated with certain peripheral vascular disorders that can lead to arterial constriction and erythema over the hypothenar eminence [42]. The nerve may be persistently entrapped due to the presence of an accessory abductor digiti minimi muscle (Figure 18B) [42].

#### 3.2.3. Dorsal Ulnar Cutaneous Nerve

##### Anatomy

The dorsal ulnar cutaneous nerve branches from the main trunk at the distal ulnar aspect of the forearm, coursing initially beneath the FCU tendon. After piercing the antebrachial fascia, it reaches the dorsal aspect of the wrist, and then divides into common digital branches to provide innervation to the little finger and ulnar side of the ring finger.

##### Scanning Technique

**Figure 19:**
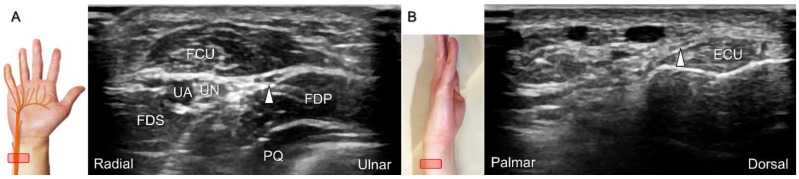
Sonographic imaging of the dorsal ulnar cutaneous nerve as it branches from the ulnar aspect of the ulnar nerve underneath the flexor carpi ulnaris (FCU) muscle (**A**). The nerve wraps around the distal ulna to reach the dorsal wrist (**B**). Arrowhead: dorsal ulnar cutaneous nerve. UN: ulnar nerve; UA: ulnar artery; FDS: flexor digitorum superficialis muscle; FDP: flexor digitorum profundus muscle; PQ; pronator quadratus muscle; ECU: extensor carpi ulnaris tendon.

With the forearm in supination, the transducer is placed on the distal third of the ventral forearm to locate the myotendinous junction of the FCU. The dorsal ulnar cutaneous nerve branches from the ulnar aspect of the ulnar nerve underneath the FCU, and then wraps around the distal ulna to reach the dorsal wrist. Moving the transducer to the dorsal wrist, the nerve can be seen coursing above the extensor carpi ulnaris tendon (Figure 19), toward the dorsal ulnar wrist and hand region.

##### Clinical Implication

**Figure 20:**
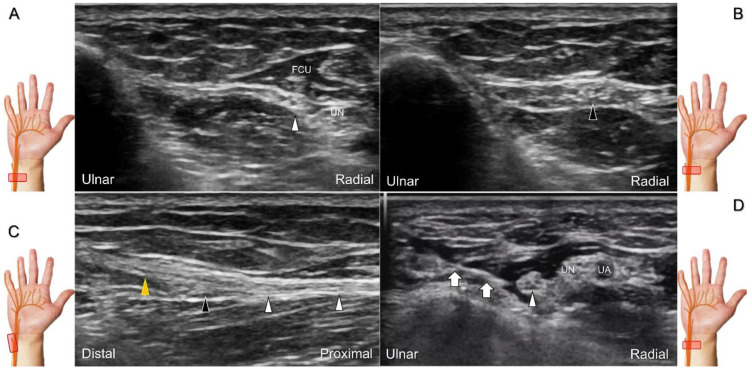
Sonographic tracking (short-axis view) of the dorsal ulnar cutaneous nerve from its normal (**A**) to the swollen segment (**B**) proximal to the entrapment. The normal, swollen, and entrapped segments of the dorsal ulnar cutaneous nerve are seen in long-axis imaging (**C**). Ultrasound-guided hydrodissection of the nerve (**D**). White arrowheads: normal segment; black arrowheads: swollen segment; orange arrowhead: entrapped segment; white arrows: needle. FCU: flexor carpi ulnaris muscle; UN: ulnar nerve; UA: ulnar artery.

The causes of damage/entrapment of the dorsal ulnar cutaneous nerve include compression over the distal forearm by a bracelet or a metal implant, extensor carpi ulnaris tenosynovitis, or triangular fibrocartilage complex injury [43]. For hydrodissection of the entrapped nerve (Figure 20A–C), the in-plane approach targeting its short axis is preferred (Figure 20D).

### 3.3. Radial Nerve

#### 3.3.1. Superficial Radial Nerve

##### Anatomy

The superficial radial nerve is a branch of the radial nerve, originating from the posterior cord of the brachial plexus. It descends underneath the brachioradialis muscle and courses lateral to the radial artery. Upon reaching the radial aspect of the distal forearm, it perforates the antebrachial fascia, and travels between the brachioradialis and extensor carpi radialis longus tendons. It then courses to the dorsal wrist between the first and second dorsal extensor compartments, where it divides into two branches. The dorsolateral branch supplies the sensation of the dorsal radial region of the thumb proximal to the interphalangeal joint. The dorsomedial branch innervates the dorsoulnar aspect of the thumb proximal to the interphalangeal joint, dorsoradial half of the hand and the index, middle, and radial half of the ring fingers, proximal to the distal interphalangeal joint.

##### Scanning Technique

**Figure 21:**
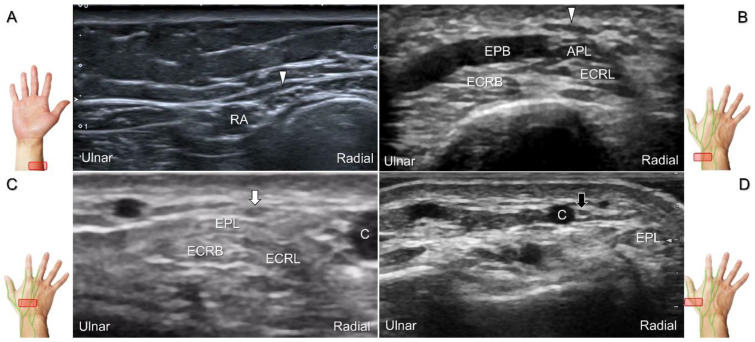
Sonographic imaging (short-axis view) of the superficial radial nerve from the distal third of the supinated forearm (**A**). With the pronated forearm, the nerve is seen to travel above the proximal intersection junction (**B**), divide into the dorsomedial branch coursing above the distal intersection junction (**C**), and the dorsolateral branch running beside the extensor pollicis longus tendon (**D**). Arrowheads: superficial radial nerve; white arrow: dorsomedial branch; black arrow: dorsolateral branch. RA: radial artery; APL: abductor pollicis longus tendon; EPB: extensor pollicis brevis tendon; ECRL: extensor carpi radialis longus tendon; ECRB: extensor carpi radialis brevis tendon; EPL: extensor pollicis longus tendon; C: cephalic vein.

With the forearm supinated, the transducer is positioned in the axial plane at the lateral aspect of the antecubital fossa. The superficial and deep radial nerves are situated between the brachioradialis and brachialis muscles. The superficial radial nerve initially courses next to the radial artery below the brachioradialis muscle, and then departs from the radial artery in the distal third of the forearm (Figure 21A). Distally, it pierces the antebrachial fascia between the extensor carpi radialis longus and brachioradialis tendons. When tracking the terminal portion of the superficial radial nerve, the forearm can be pronated, as it courses toward the dorsal radial aspect of the wrist/hand. The superficial radial nerve travels above the proximal intersection junction between the first and second dorsal extensor compartments (Figure 21B). Later, it divides into the dorsomedial and dorsolateral branches. The former courses above the distal intersection junction of the second and third compartments (Figure 21C). The latter runs beside the extensor pollicis longus tendon (Figure 21D).

##### Clinical Implication

**Figure 22:**
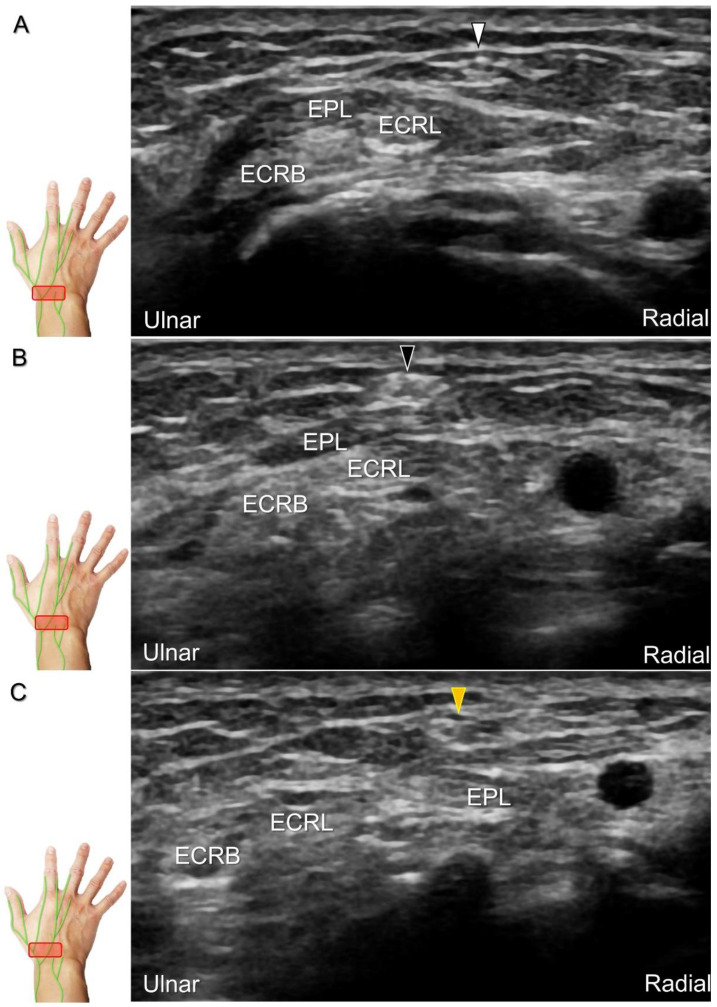
Sonographic imaging (short-axis view) of the dorsolateral branch of the superficial radial nerve at normal (**A**), swollen (**B**), and compressed (**C**) segments. White arrowhead: normal segment; black arrowhead: swollen segment; orange arrowhead: entrapped segment. ECRL: extensor carpi radialis longus tendon; ECRB: extensor carpi radialis brevis tendon; EPL: extensor pollicis longus tendon.

Cheiralgia paresthetica, also known as Wartenberg’s syndrome, is the compressive neuropathy of the superficial radial nerve. Symptoms such as tenderness, numbness, and allodynia can be exacerbated by wrist flexion and ulnar deviation. The nerve can be compressed by a handcuff, watch, bracelet, metal implant, ganglion cyst, or distal radius fracture. Nerve entrapment commonly takes place at the proximal intersection zone pertaining to the first and second extensor compartments (Figure 22).

**Figure 23:**
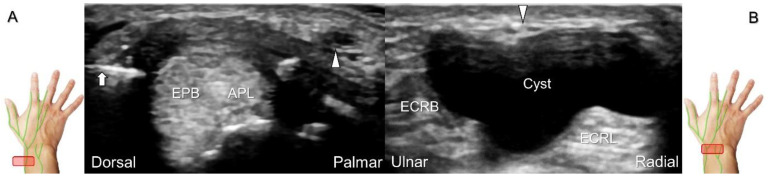
Sonographic imaging of the superficial radial nerve beside the first extensor compartment of the wrist (**A**), and a ganglion cyst over the second extensor compartment of the wrist (**B**). White arrowheads: superficial radial nerve; white arrow: needle. APL: abductor pollicis longus tendon; EPB: extensor pollicis brevis tendon; ECRL: extensor carpi radialis longus tendon; ECRB: extensor carpi radialis brevis tendon.

**Figure 24:**
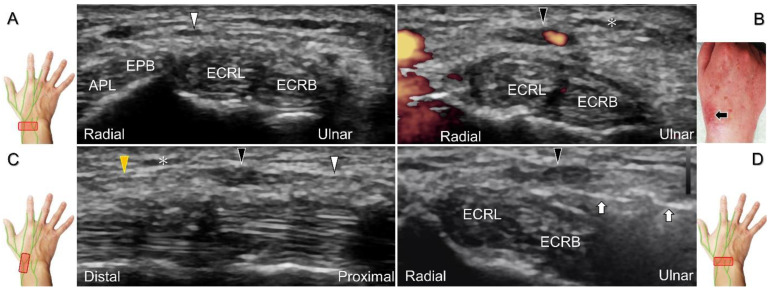
Sonographic imaging of the superficial radial nerve entrapment due to a post-surgical scar. Short-axis imaging at the normal (**A**) and the swollen (**B**) segment proximal to the entrapment. The normal, swollen, and entrapped segments of the nerve are seen in long-axis view (**C**). Ultrasound-guided hydrodissection of the nerve from the ulnar aspect (**D**). White arrowheads: normal segment; black arrowheads: swollen segment; orange arrowhead: entrapped segment; white arrows: needle; black arrow: scars on the skin; asterisk: scars in the subcutaneous tissue; APL: abductor pollicis longus tendon; EPB: extensor pollicis brevis tendon; ECRL: extensor carpi radialis longus tendon; ECRB: extensor carpi radialis brevis tendon.

Additionally, acupuncture and cannulation of the cephalic vein over the distal forearm can lead to nerve injury. To prevent iatrogenic injury, the superficial radial nerve before injection should also be recognized for de Quervain’s syndrome (Figure 23A) [44], ganglion cyst aspiration (Figure 23B), and catheterization (Figure 24A–C). For injection of the superficial radial nerve, an in-plane approach in the nerve’s short axis is preferred (Figure 24D).

**Figure 25:**
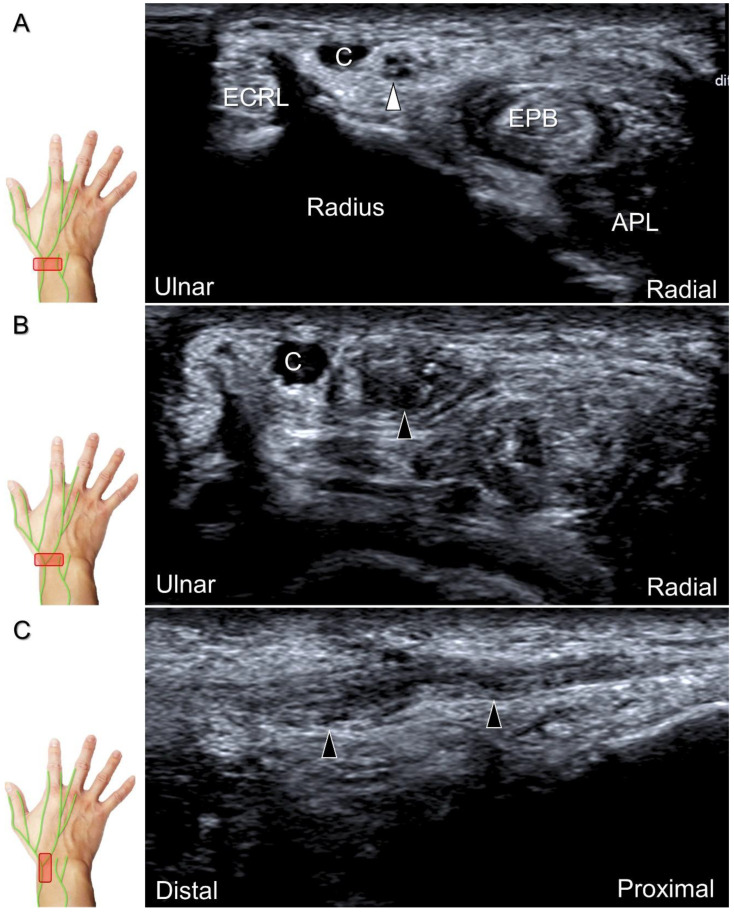
Sonographic images depict the location of a neuroma of the superficial radial nerve. The normal/proximal segment (**A**) and the neuroma in short-axis (**B**) and long-axis (**C**) views are seen. White arrowhead: superficial radial nerve; black arrowheads: neuroma; APL: abductor pollicis longus tendon; EPB: extensor pollicis brevis tendon; ECRL: extensor carpi radialis longus tendon; C: cephalic vein.

For those receiving surgery such as fixation or debridement of the radial wrist, the nerve can be occasionally injured, resulting in residual numbness, allodynia, dysesthesia, or hypoesthesia (Figure 25).

#### 3.3.2. Posterior Interosseous Nerve

##### Anatomy

The posterior interosseus nerve is initially located at the radial aspect of the proximal forearm, situated between the brachioradialis and brachialis muscles with the superficial radial nerve. It then passes under the arcade of Frohse, which is the superficial layer of the supinator muscle. Later, it courses through the radial tunnel formed by the humeral and ulnar head of the supinator muscle and continues between the extensor digitorum longus and abductor pollicis longus (or extensor pollicis brevis) muscles before diving to run on top of the interosseous membrane, a fibrous tissue that connects the radius to the ulna [45]. The posterior interosseus nerve provides motor innervation to all dorsal forearm muscles except the brachioradialis, anconeus, and extensor carpi radialis longus. It also provides proprioception to the dorsal radioulnar joint.

##### Scanning Technique

**Figure 26:**
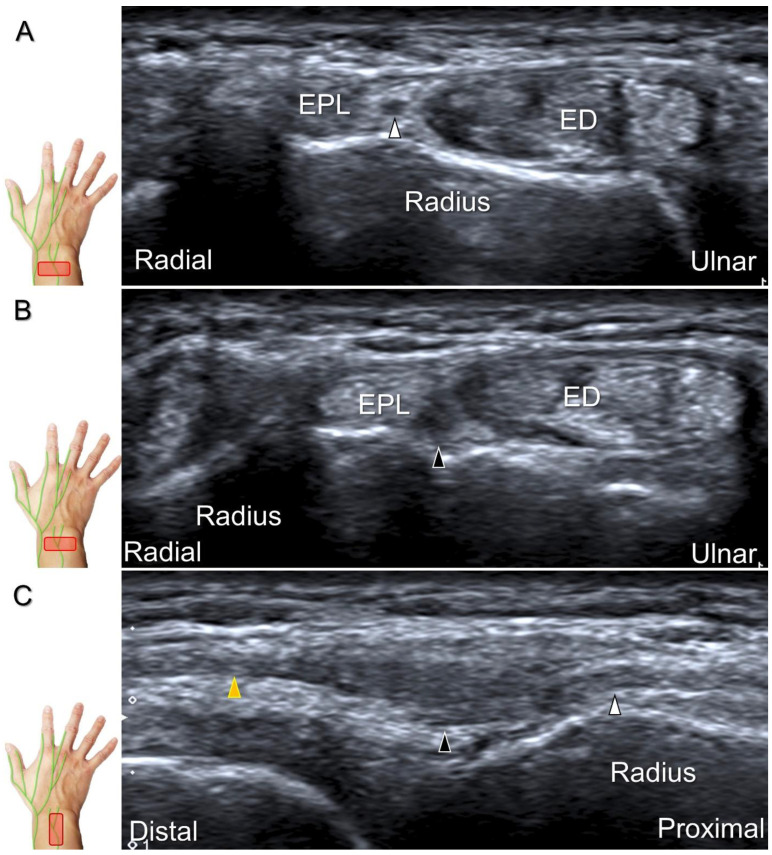
Sonographic imaging (short-axis view) of the dorsal interosseus nerve from the normal (**A**) to the swollen (**B**) segment proximal to the entrapment site. Normal, swollen, and compressed segments of the nerve are seen in long-axis view (**C**). White arrowhead: normal segment; black arrowhead: swollen segment; yellow arrowhead: entrapped segment; EPL: extensor pollicis longus tendon; ED: extensor digitorum tendon.

With the forearm supinated, the transducer is placed at the radial side of the upper third of the forearm in short-axis view. The posterior interosseus nerve is situated between the extensor digitorum longus and abductor pollicis longus (or extensor pollicis brevis) muscles. The transducer is moved distally along the dorsal forearm. The nerve courses radial to the extensor pollicis longus muscle and then dives toward the surface of the dorsal interosseous membrane. At the wrist level, the nerve appears as a small hypoechoic monofascicle with an average diameter about 1–3 mm [46] and then it travels onto the carpus with the dorsal interosseous artery (Figure 26A).

##### Clinical Implication

**Figure 27:**
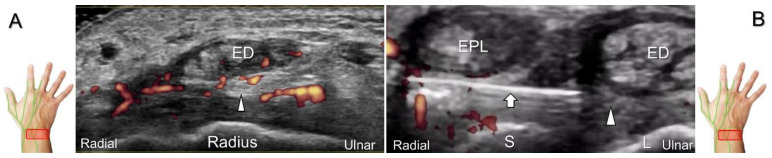
Sonographic imaging (short-axis view) is performed to assess the posterior interosseus nerve entrapment due to synovitis in rheumatoid arthritis (**A**). Ultrasound-guided injection (**B**). Arrowheads: posterior interosseus nerve; arrow: needle; EPL: extensor pollicis longus tendon; ED: extensor digitorum communis tendon; S: scaphoid; L: lunate.

Nerve entrapment can occur from repetitive and forceful use of extensor digitorum longus and extensor pollicis longus muscles. Contusion to the wrist can traumatize the posterior interosseus nerve, leading to neuroma formation (Figure 26B,C). The nerve can also be irritated by hypertrophic synovium secondary to extensor digitorum communis tenosynovitis (Figure 27A) [47]. When injecting the scapho-lunate joint, the nerve should be identified first to avoid iatrogenic injury. In cases suffering from pain or allodynia over the dorsal radioulnar joint, hydrodissection of the nerve can be performed. The in-plane approach during the nerve’s short-axis view is the preferred method (Figure 27B) whereby iatrogenic injury of the vessels and extensor tendons can be prevented.

### 3.4. Digital Nerves

#### 3.4.1. Palmar Common Digital and Proper Digital Nerves

##### Anatomy

The palmar common digital nerves arise from two sources; the median nerve, which supplies sensation to the palmar aspect of all phalanges of the first, second, third, and radial aspect of the fourth digit, and the superficial branch of the ulnar nerve, which supplies the remaining palmar aspect of the phalanges. The palmar common digital nerves then give rise to the proper digital nerves that course along the bilateral palmar sides of the digits. Furthermore, the palmar proper digital nerves—which receive fibers from the median nerve—provide sensory innervation to the dorsal aspect of the middle and distal phalanges of the second, third, and radial aspect of the fourth finger.

##### Scanning Technique

**Figure 28:**
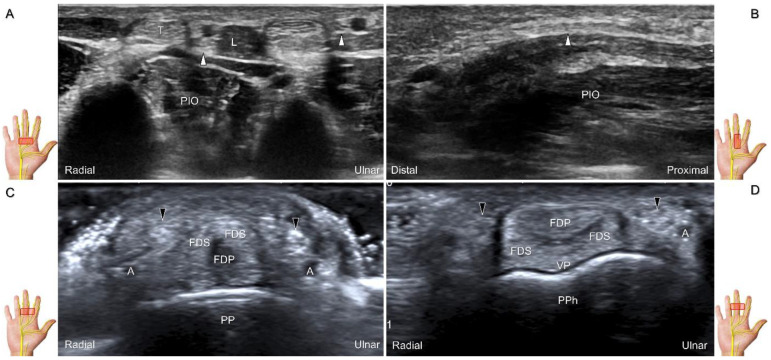
Sonographic imaging of the palmar common digital nerves in short-axis (**A**) and long-axis (**B**) views. Palmar proper digital nerves from the base (**C**) to the head of the proximal phalanx (**D**). White arrowheads: palmar common digital nerves; black arrowheads: palmar proper digital nerves. T: flexor tendon; L: lumbricalis muscle; PIO: palmar interosseous muscle; FDS: flexor digitorum superficialis tendon; FDP: flexor digitorum profundus tendon; A: artery; VP: volar plate; PP: proximal phalanx; PPh: head of the proximal phalanx.

The transducer is placed on the mid-palm in the axial plane (Figure 28A). The palmar common digital nerves course beside the flexor digitorum profundus/superficialis tendons with the palmar common digital artery, and they are superficial to the palmar interosseous muscles (Figure 28B). Moving the transducer more distally, the palmar proper digital nerves can be identified alongside all phalanges (Figure 28C,D).

##### Clinical Implication

**Figure 29:**
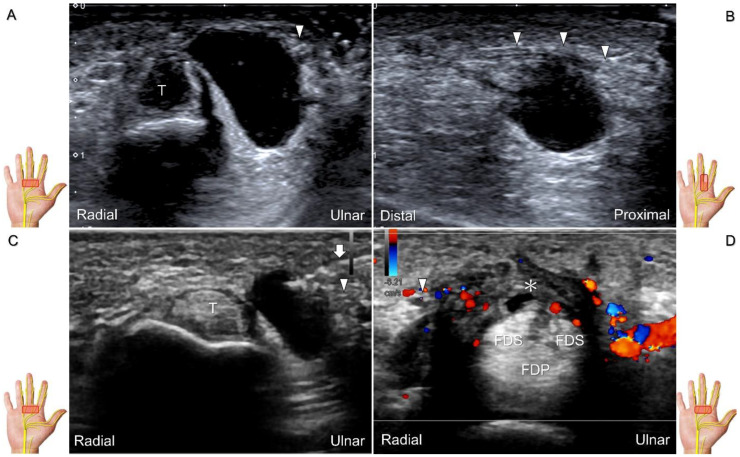
Ultrasound images demonstrate the entrapment of the palmar common digital nerve due to a ganglion seen in short-axis (**A**) and long-axis (**B**) views. Ultrasound-guided aspiration (**C**). Nerve entrapment due to annular ligament tear (asterisk) following an iatrogenic injury (**D**). Arrowheads: palmar common digital nerve; Arrow: needle; T: flexor tendon; FDS: flexor digitorum superficialis tendon; FDP: flexor digitorum profundus tendon.

**Figure 30:**
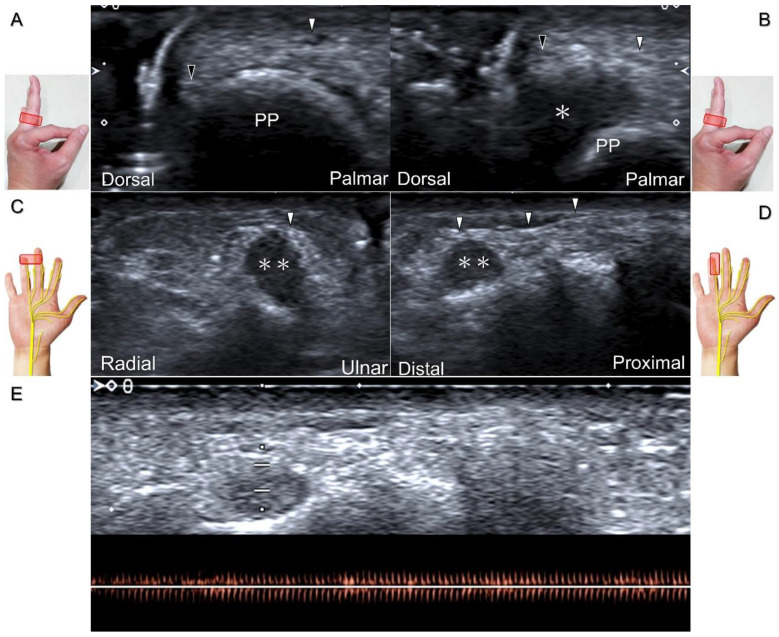
In comparison to the healthy side (**A**), short-axis imaging (**B**) shows irritation of both the palmar and dorsal proper digital nerves due to a fibroma (asterisk). Short-axis (**C**) and long-axis (**D**) imaging demonstrates irritation of the palmar proper digital nerve due to a hemangioma. Spectral Doppler mode (**E**) confirms the hemangioma. White arrowhead: palmar proper digital nerve; black arrowhead: dorsal proper digital nerve; double asterisk: hemangioma; PP: proximal phalanx.

Direct injury of the palmar common digital nerve can occur due to various reasons such as trauma, contusion, or iatrogenically during tendon injection. Nerve entrapment caused by space-occupying lesions such as fractures, ganglia (Figure 29A–C), annular ligament tears (Figure 29D), tenosynovitis, foreign bodies, fibroma (Figure 30A,B), or hemangioma (Figure 30C–E) is also likely.

**Figure 31:**
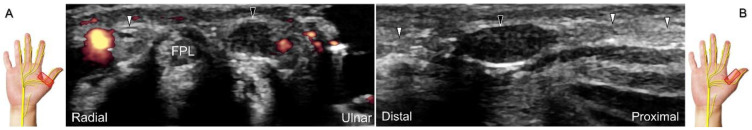
Sonographic imaging of the neuroma of the palmar proper digital nerve in short-axis (**A**) and long-axis (**B**) views. White arrowheads: normal segments of the palmar proper digital nerve; black arrowhead: neuroma. FPL: flexor pollicis longus tendon.

**Figure 32:**
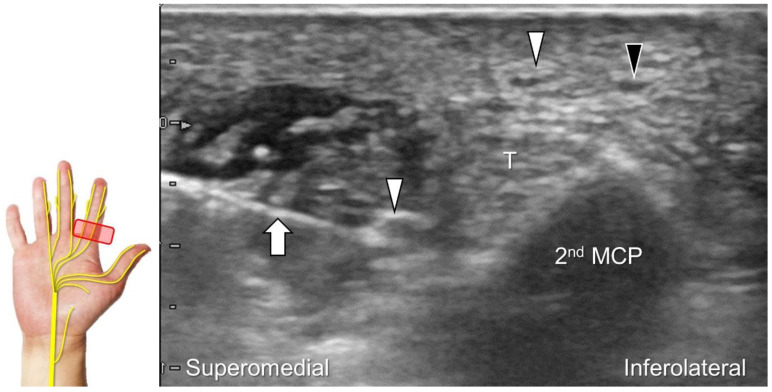
In-plane ulnar to radial approach is used for injecting the palmar common digital nerve in short-axis view. White arrowheads: palmar common digital nerve; black arrowhead: common palmar digital artery; arrow: needle; T: flexor tendons; 2nd MCP: second metacarpal.

Chronic irritation may lead to the formation of a neuroma, which can occasionally be detected through US imaging (Figure 31). Using the in-plane approach in short-axis view (Figure 32, Video S4), hydrodissection of the entrapped nerves can be performed after identifying the palmar common digital artery and flexor digitorum superficialis/profundus tendons.

#### 3.4.2. Dorsal Common Digital Nerve and Proper Digital Nerve

##### Anatomy

The dorsal common digital nerves arise from two primary nerves; the superficial radial nerve and the dorsal ulnar cutaneous nerve. The former provides sensory innervation to the dorsolateral aspect of the wrist, the dorsal thumb proximal to the distal phalanx, the proximal phalanx of the second digit, and the radial aspect of the third digit. Additionally, the dorsal ulnar cutaneous nerve supplies sensory innervation to the dorsomedial aspect of the wrist, the dorsal little finger, the ulnar aspect of the fourth digit, the proximal phalanx of the radial aspect of the fourth digit, and the ulnar aspect of the third digit. At the digital level, the dorsal common digital nerves divide into the dorsal proper digital nerves, which run superficial to the extensor expansion that originates from the dorsal interossei and lumbricalis muscles.

##### Scanning Technique

**Figure 33:**
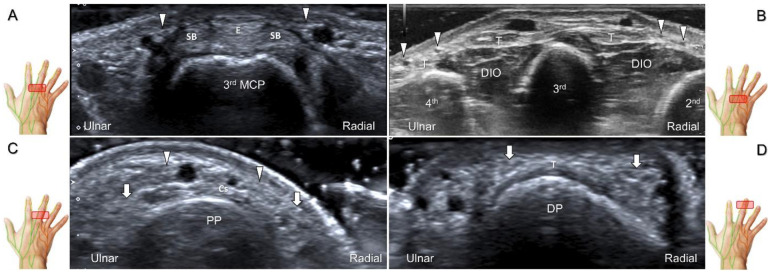
Sonographic imaging of the dorsal common digital nerve on the metacarpal bone (**A**), toward the metacarpal head (**B**). Dorsal proper digital nerves on the proximal phalanx (**C**), and the terminal nerve originating from the palmar proper digital nerve on the distal phalanx of third finger (**D**). Arrowheads: dorsal common digital nerve; arrows: palmar proper digital nerve. MCP: metacarpal bone; SB: sagittal band; E: extensor tendon; Cs: central slip; PP: proximal phalanx; T: terminal band; DP: distal phalanx; DIO, dorsal interosseous muscle.

The transducer is positioned on the axial plane of the dorsal metacarpal joint in the target digit. The dorsal proper digital nerves can be found on either side of the sagittal band (Figure 33A). The transducer is moved proximally and the dorsal common digital nerves can be observed superficial to the extensor (usually the extensor digitorum proprius) tendons (Figure 33B). By returning to the level of the sagittal band and moving the transducer distally, the dorsal proper digital nerves can be seen superficial to and alongside the central slip of the finger extensor tendon (Figure 33C). Alternatively, moving the transducer distally toward the proximal phalanx of the first to the radial aspect of the fourth phalanx allows observation of the nerve fascicles of the palmar proper digital nerve originating from the median nerve (Figure 33D).

##### Clinical Implication

**Figure 34:**
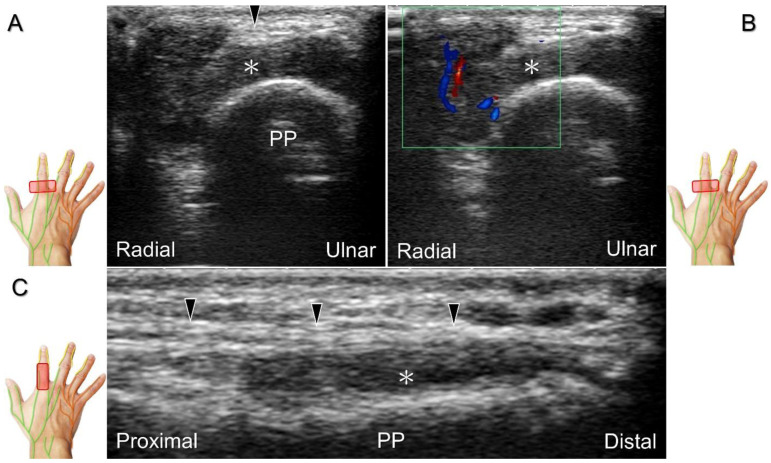
In the short-axis view, the proper digital nerve is seen as irritated by a giant cell tumor (**A**) with increased vascular signals (**B**). The association between the proper digital nerve and the tumor is delineated in the long-axis view (**C**). Black arrowheads: proper digital nerve; PP: proximal phalanx; *: giant cell tumor.

**Figure 35:**
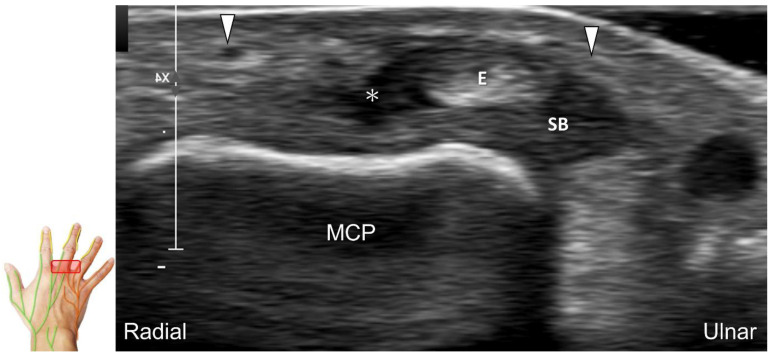
Sonographic imaging reveals irritation of the ulnar aspect of the dorsal common digital nerve due to extensor tendon subluxation resulting from a tear in the sagittal band. Arrowheads: dorsal common digital nerve; asterisk: tear of the sagittal band. SB: sagittal band; E: extensor tendon; MCP: metacarpal bone.

Injuries to the dorsal common digital nerve typically occur in the workplace as a result of cutting or crushing. However, the nerve can also sustain damage due to various other factors such as fracture, ganglia, tenosynovitis, tumor (Figure 34), foreign objects, or boxing, which may cause contusion over the first knuckles (Figure 35).

**Figure 36:**
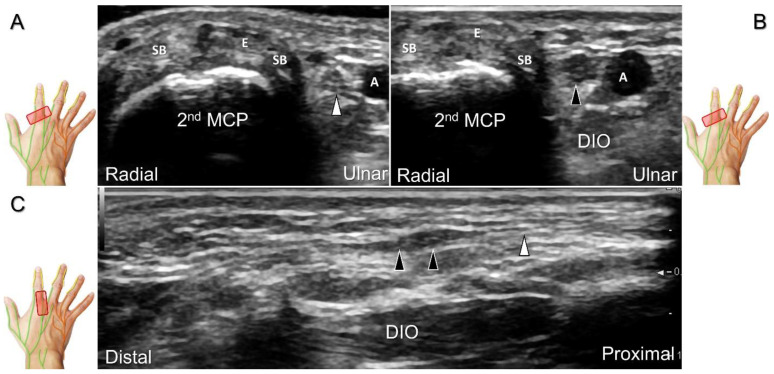
Sonographic imaging (short-axis view) for a neuroma of the dorsal common digital nerve, proximal site (**A**) and the site of the lesion (**B**). Long-axis imaging of the nerve/neuroma (**C**). White arrowhead: normal dorsal common digital nerve; black arrowheads: neuroma. SB: sagittal band; E: extensor tendon; MCP: metacarpal bone; DIO: dorsal interosseous muscle; A: artery.

**Figure 37:**
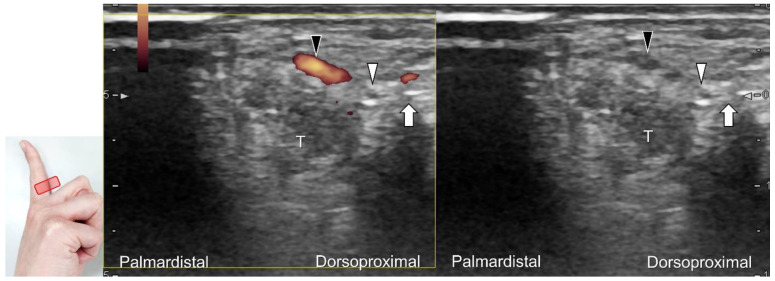
Ultrasound-guided injection to the dorsal proper digital nerve in its short axis with the dual imaging mode (Doppler vs. B mode). White arrowhead: dorsal proper digital nerve; black arrowhead: dorsal proper digital artery; arrow: needle. T: flexor tendons.

US imaging may reveal a neuroma in cases where patients report chronic allodynia and/or tingling sensation in the affected digit (Figure 36). To perform hydrodissection, the in-plane approach can be utilized in the nerve’s short axis after identifying the extensor digitorum tendons and the dorsal metacarpal arteries (Figure 37).

## 4. Conclusions

This pictorial essay outlines a systematic US scanning approach for examining the distal nerves in the wrist/hand region. US examination can assist to identify/describe the affected nerves and their morphological changes. Sonographic findings can also complement electrodiagnostic studies to provide better insight into understanding the whole clinical scenario. Needless to say, US-guided interventions (as appropriate) can be safe and effective for treating relevant nerve pathologies. Lastly, further research can be conducted to compare the diagnostic performance of US imaging with other tools such as MRI and electrophysiological testing.

## Data Availability

Data are contained within the main text of the manuscript.

## Supplementary Materials

The following supporting information can be downloaded at: https://www.mdpi.com/article/10.3390/diagnostics13111928/s1, Video S1: Hydrodissection with the in-plane approach targeting the short axis of the median nerve. Video S2: Hydrodissection with the in-plane approach targeting the long axis of the median nerve. Video S3: Dynamic tracking of the neuroma of recurrent motor branch of the median nerve. Video S4: Hydrodissection using in-plane approach targeting the short axis of the palmar common digital nerve of the thumb.
